# Supplementary material for: Upright walking has driven unique vascular specialization of the hominin ilium
Source: PeerJ. 2021 Oct 19;9:e12240. doi: 10.7717/peerj.12240 (PMC8532992; doi:10.7717/peerj.12240)
Supplement: Supplemental Information 1 [file peerj-09-12240-s001.docx]

**SUPPLEMENT 1: IDENTIFICATON OF THE VASCULAR CONTENTS OF THE AFI**

In order to potentially identify any arteries associated with the AIF we dissected three adult human cadavers (2 females, 1 male). In all three specimens the *ascending branch* of the Lateral Circumflex Femoral Artery (LCFA) lay in close proximity to the AIF. This is consistent with previous reports that this vessel regularly rests on the ilium’s anterolateral surface (Xu & Zhong, 1989). In our specimens the LCFA and its associated vein ran together within a thin sheath for a portion of their length. The vein of the ascending branch joined the artery in this sheath just prior to passing beneath the rectus femoris muscle. The LCFA then ascended further to reach the lateral ilium very near the regular position of the AIF.

Some sources report that the superficial and deep circumflex iliac arteries course near this general region and occasionally anastomose with an ascending branch of the LCFA (Williams, 1995; Rehman & Smith, 1980). However, in our specimens both the superficial and deep iliac circumflex arteries were located too far superiorly nearer the iliac crest to do so and did not appear to be directly associated with the AIF or the LCFA/V, which appear likely to be the occupants of the AIF.

We also examined CT scans of living subjects. In one we were able to identify a branch of the LCFA/V that entered the AIF on the right side, although one from the Superior Gluteal Artery (SGA) entered the left AIF (Supp. Figure 1). Williams (1995) reports that the SGA “in the pelvis…supplies the piriformis, obturator internus, and *an* *innominate nutrient artery”* (p.1562: emphasis added). It would seem almost certain that the authors were describing this lateral arterial vessel as the contents of the AIF, and not the primary nutrient vessel (i.e., the NFI) that lies more medially, as noted earlier.

Branches of the SGA and the LCFA anastomose in the general vicinity of the lateral ilium (Al-Talalwah, 2015). The SGA emerges from a posterior branch of the internal iliac a. and the LCFA usually arises from the deep femoral a., which is a branch of the femoral artery. The latter is a continuation of the external iliac a. Based on these findings collectively, we conclude that either a subsidiary of the ascending branch of the LFCA/V and/or one from the SGA is a routine arterial occupant of the AIF.


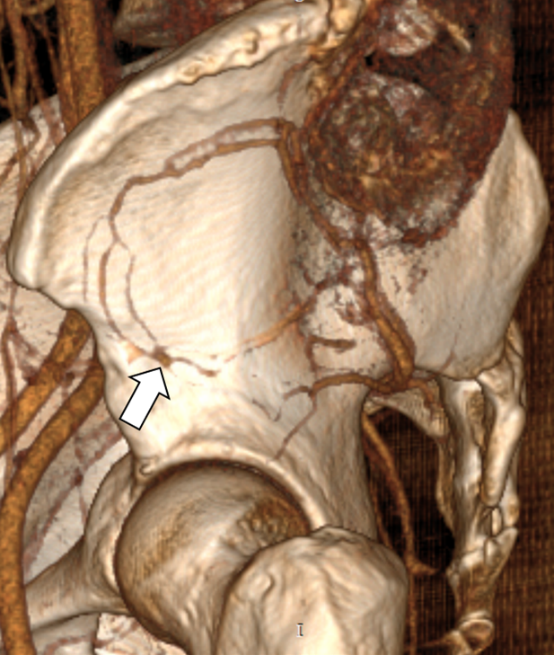


**Supplemental Fig. 1.** Digital reconstruction of the left lateral pelvis displaying extensions of the superior gluteal arteries/veins clearly inserting into a very large AIF (arrow). Male 42 years, from sarcoma archives of The Cancer Imaging Archive (TCIA), with permission. This image is a reconstruction and window we generated that was derived from a CT scan within TCIA. The results shown here are in whole or part based upon data in generated by the TCGA Research Network: <http://cancergenome.nih.gov/>. See also: Roche, Bonaccio, & Filippini (2016), and Clark et al. (2013).

Xu, D.; Kong, J.; Zhong, S. (1989). The ascending branch of the lateral circumflex femoral artery. *Surgical and Radiologic Anatomy*, 11 (4), 263-264.

Williams, P.L. (1995). Gray’s anatomy. New York: Churchill Livingstone, 749-789.

Rehman, I.; Smith, C. F.(1980). Illustration of Left Thigh, Anterio-Lateral View, Showing Muscles, Bone, and Artery (Deep Circumflex Iliac). In Orthopaedic Surgical Anatomy Teaching Collection, Norris Medical Library, USC Digital Library: University of Southern California, Modified: 1990/2000.

Al-Talalwah, W. (2015). The Vascular Supply of Hip Joint and its Clinical Significant. *International Journal of Morphology*, 33 (1).

Roche, C., Bonaccio, E., & Filippini, J. (2016). Radiology Data from The Cancer Genome Atlas Sarcoma [TCGA-SARC] collection. The Cancer Imaging Archive. <http://doi.org/10.7937/K9/TCIA.2016.CX6YLSUX>

Clark K., Vendt B., Smith K., Freymann J., Kirby J., Koppel P., Moore S., Phillips S., Maffitt D., Pringle M., Tarbox L., Prior F. (2013). *The Cancer Imaging Archive* (TCIA): Maintaining and Operating a Public Information Repository, Journal of Digital Imaging, Volume 26, Number 6, December, pp 1045-1057.

**SUPPLEMENT 2: Non-hominoid specimens examined for the presence of regional foramina of the anterior ilium**


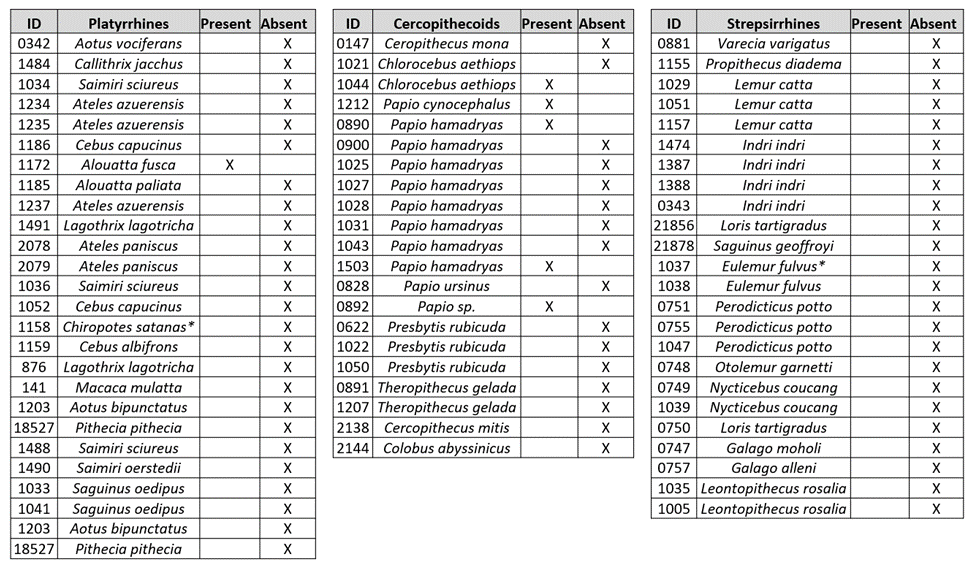


**
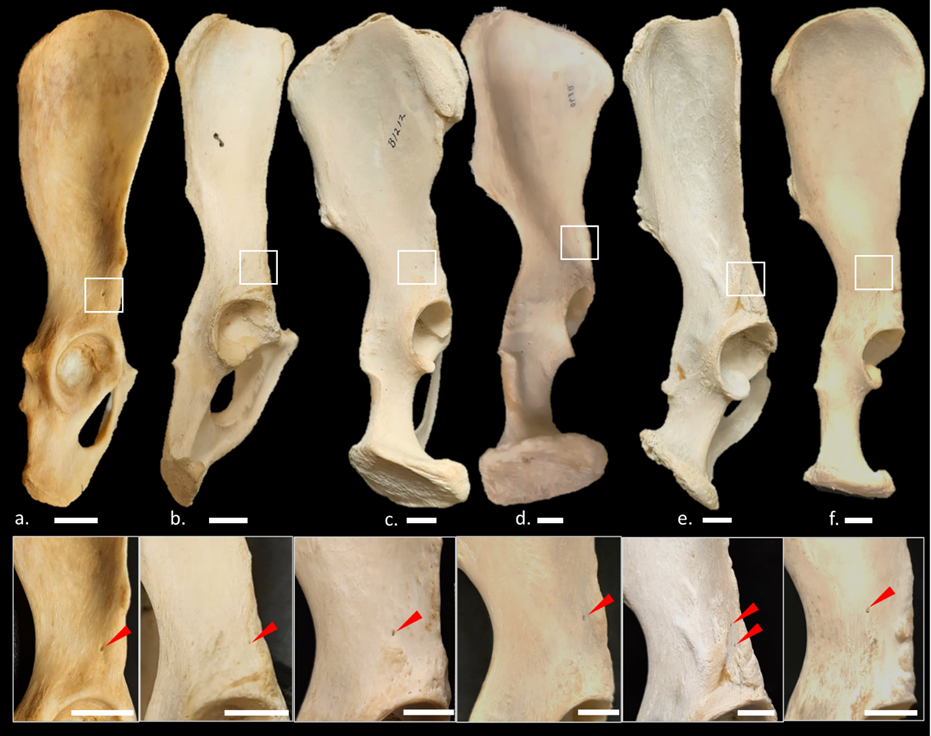
**

**Supplemental Figure 2: Regional Iliac Foramina Among New World and Old World Monkeys.**

Select specimens in which regional foramina of the anterior ilium were observed (red arrows). Foramina of the anterior ilium were exceedingly rare in the New World and Old World monkeys that were evaluated (see S5). A regional “candidate” iliac foramen was detected in only one side (left or right) in each specimen in which it was found, i.e., no cases of bilateral foramina were found in our survey of these non-hominoid specimens. Additional sizeable foramina of the ischium were occasionally observed (yellow arrows). Specimens: **(a)** *Alouatta fusca* (1172), **(b)** *Chlorocebus aethiops* (1044)*,* **(c)** *Papio cynocephalus* (1212), **(d)** *Papio hamadryas* (0890, image reversed), **(e)** *Colobus abyssinicus* (18525), **(f)** *Papio sp.*(0892, image reversed). Scale bars = 1 cm.

**
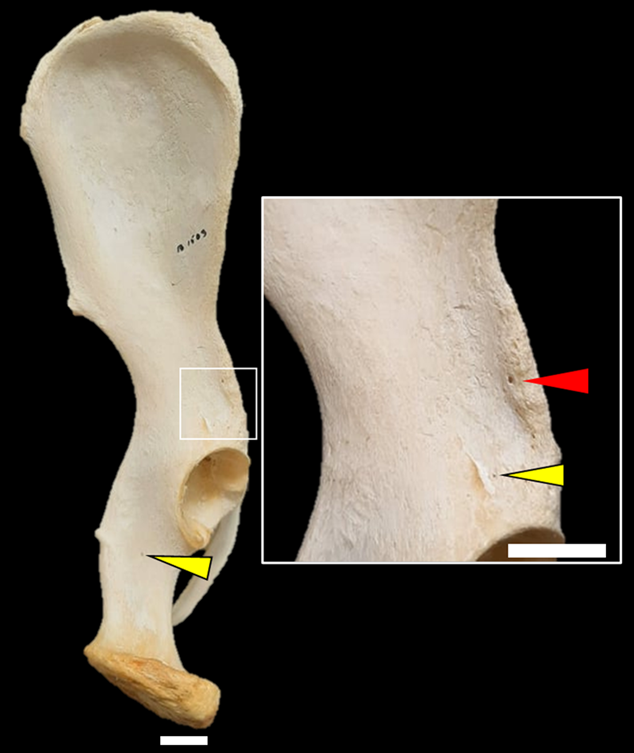
**

**Supplemental Figure 3. Regional Iliac Foramina of *Papio hamadryas* (1503)*.***

Foramina of the anterior ilium (red arrow), superior acetabular rim, and ischium (yellow arrows). Inset displaying close-up of the iliac isthmus and respective foramina. Scale bar = 1 cm.

**SUPPLEMENT 3:** Vascular Canal System in Ilia of Humans and Extant Great Apes (*H. sapiens, G. gorilla, P. troglodytes, P. pygmaeus*). Fiber Passage: Successful (S) /Unsuccessful (U).

| ***Homo*** | **Result** |  | ***Gorilla*** | **Result** |  | ***Pan*** | **Result** |  | ***Pongo*** | **Result** |
| --- | --- | --- | --- | --- | --- | --- | --- | --- | --- | --- |
| 31200 | S |  | 3557 | U |  | 1056 | U |  | 623 | U |
| 04007 | S |  | 3556 | U |  | 629 | U |  | 1030 | U |
| 01001 | S |  | 3549 | U |  | 1726 | U |  | 1055 | U |
| 00206 | S |  | 3548 | U |  | 1723 | U |  | 1167 | U |
| 04035 | S |  | 3547 | U |  | 1721 | U |  | 1168 | U |
| 02208 | S |  | 3546 | S |  | 1766 | U |  | T/W 15-8 | U |
| 04005 | S |  | 3405 | U |  | 1762 | U |  |  |  |
| 04026 | S |  | 3404 | U |  | 1758 | U |  |  |  |
| 02247 | S |  | 3400 | U |  | 1755 | U |  |  |  |
| 03204 | S |  | 2829 | U |  | 1749 | U |  |  |  |
| 03237 | S |  | 2826 | U |  | 1880 | U |  |  |  |
| 02252 | S |  | 2775 | U |  | 2823 | U |  |  |  |
| 02043 | U* |  | 2767 | U |  | 2771 | U |  |  |  |
| 00237 | S |  | 2742 | U |  | 2746 | U |  |  |  |
| 00055 | S |  | 2741 | U |  | 3552 | U |  |  |  |
| 01272 | S |  | 2739 | U |  | 1433 | U |  |  |  |
| 02206 | S |  | 1408 | U |  | 1434 | U |  |  |  |
| 09017 | U* |  | 1407 | U |  | 1707 | U |  |  |  |
| 04044 | U* |  | 1057 | U |  | 1708 | U |  |  |  |
| 04041 | S |  | 1710 | U |  | 1713 | U |  |  |  |
| 05030 | S |  | 1709 | U |  | 1735 | U |  |  |  |
| 01225 | S |  | 1704 | U |  | 1737 | U |  |  |  |
| 00213R | S |  | 1431 | U |  | 1738 | U |  |  |  |
| 01010 | S |  | 1430 | U |  | 1775 | U |  |  |  |
| 00254 | S |  | 1425 | U |  | 1770 | U |  |  |  |
| 01004 | U* |  | 1736 | U |  | 1769 | U |  |  |  |
| 02025 | S |  | 1733 | S |  | 2027 | U |  |  |  |
| 32218 | S |  | 1731 | U |  | 2026 | U |  |  |  |
| 04095 | S |  | 1730 | U |  |  |  |  |  |  |
| 04023 | U* |  | 1729 | U |  |  |  |  |  |  |
| 00739 | S |  | 1756 | U |  |  |  |  |  |  |
| 03001 | S |  | 1801 | U |  |  |  |  |  |  |
| 02036 | S |  |  |  |  |  |  |  |  |  |
| 02609 | U* |  |  |  |  |  |  |  |  |  |
| 05011 | S |  |  |  |  |  |  |  |  |  |
| 02217 | U* |  |  |  |  |  |  |  |  |  |
| 04044 | S |  |  |  |  |  |  |  |  |  |
| 00213L | S |  |  |  |  |  |  |  |  |  |

*Note: Specimens 09017 & 04044: Partially impacted with sediment. 01004: The fiber has limited capacity to account for internal deviations from the standard linear path. Orientation of the AIIS appeared to restrict fiber passage through the AIF in this specimen. 04023: The nutrient foramen canal bifurcated approximately 3 mm internally, thus producing two canals that were too small to allow further fiber passage. This bifurcation was visually confirmed by shining a light source into the canal. This specimen also displayed some sediment obstruction. 02609: The fibers traversed the appropriate length for each to make possible contact but attempts to produce motion by contact between the two fibers were unsuccessful. It is possible that each followed opposite trabecular walls (NF medially, AIF laterally). The fiber cannot account for a possible internal transverse connections or paths outside the primary semi-linear canal path. 02043 & 02217: Fibers were unsuccessful in these specimens, but we could not identify the cause. Plausible explanations in any of the Libben specimens is the presence of excessive sediment as the interment conditions were largely fine sand and clay.

**SUPPLEMENT 4: CONSTRUCTION DETAILS OF THE PROBE USED TO MEASURE THE AFI FOR THIS STUDY**

As noted in the text, we first measured the diameter of the AIF and NFI using dental calipers. However, foramina were frequently angled and to some degree eccentric, making caliper placement sometimes problematic for guaranteed consistency, as most foramina are slightly ovoid with conically shaped entrances.

To reduce the effect of such eccentricity, we created a series of progressively size-incremented probes using “Tinkercad” software on a Formlabs Form2 3D stereolithography (SLA) printer. Build volume was 145 X 145 X 175 and resolution was 25 microns. Probe material was photopolymer resin. Each probe consists of a handle portion with a tapered tip to insert into a target foramen (see Supp. Figure 2a). Tip varies progressively from 0.5 mm to 3.0 mm in increments of 0.1 mm (see Supp. Figure 2b).

At output, because each probe was approximately 2 mm in original overall thickness, the

smallest probes in this series were hand-finished in order to assure that their tip breadths were

exact and not slightly increased by their rectangular form (i.e., a probe’s hypotenuse was

necessarily slightly increased by the probe’s original 2 mm thickness). Therefore, smaller probes

were essentially made to be only two dimensional (see Supp. Figure 2b).

Each probe was inserted into a target foramen in approximate alignment with its exit axis and allowed to conform to any eccentricity of foramen shape. The “rule” for probe conformity with each foramen was to allow it to penetrate to a depth of approximately 1.0 to 1.5 mm without

resistance, i.e., each probe was permitted to conform to slight variations in shape and angulation

in a manner that did not prevent penetration to this approximate depth. In other words, the probe

was allowed to penetrate the foramen’s underlying canal just sufficiently to ensure that its largest

dimension was being recorded. The largest probe in the series that a foramen would admit under

these strictures was considered its diameter.


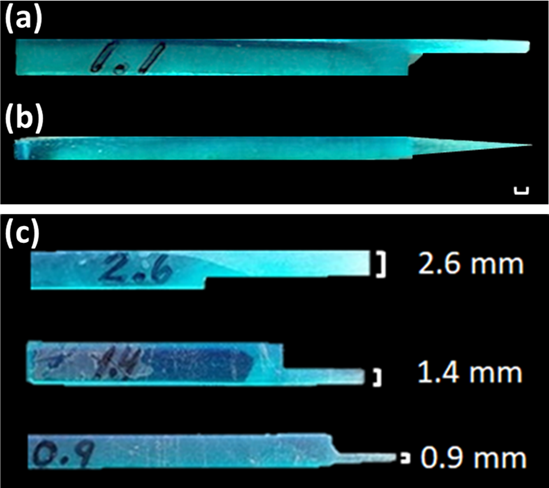


**Supplemental Fig. 4 (a)** Superior (top) and **(b)** lateral (side) views of a typical probe used in this study. As described in the text each probe is approximately 8 X 1.5 cm long and was uniformly 2 mm thick at time of production from the 3-D printer. The probe illustrated here has a 1.1 mm tip breadth and as seen in **(b)** has been thinned to a fraction of a mm so that the hypotenuse of its originally rectangular tip would not contribute significantly to its target diameter. Scale bar = 1 mm. **(c)** Same views as in **a** for examples used in cases where the foramen diameter was found to be 2.6, 1.4, and 0.9 mm. Tool diameter accuracy (following hand finishing in some cases) was = 0.1 mm. Total collection was 26 probes ranging from 0.5 - 3.0 mm tip diameter.

**SUPPLEMENT 5: HYPOTHESIS TESTS AND ESTIMATIONS FOR EACH RATIO.**

**Supplemental Table 1.** **One-Way Analysis of Variance: Index of Relative AIF Diameter in Hominoids: (AIF/NFI) *10**

Source Degrees of Sum of Mean F P
 Freedom Squares Square

Among groups 2 3444.45 1722.23 14.73 < .001

Within groups 156 18244.37 116.95

Total 158 21688.82

99% CI for Mean

Taxa n x̄ *s* Lower Upper Minimum Maximum

*H. sapiens* 65 22.01 14.91 17.63 27.34 6.32 70.00

*P. troglodytes* 46 12.66 7.58 9.80 16.37 2.78 34.00

*G. gorilla* 48 12.41 5.53 10.40 14.82 4.17 26.00

**Supplemental Table 2. One-Way Analysis of Variance: Index of Relative AIF Diameter in Hominoids: (AIF Diameter/Acetabular Diameter)*100.**

*A subset of AIF raw diameter data was used for this test of available specimens with complete acetabuli.*

Source Degrees of Sum of Mean F P
 Freedom Squares Square

Among groups 2 56.2188 28.1094 28.40 < .001

Within groups 117 115.7998 .9897

Total 119 172.0187

99% CI for Mean

Taxa n x̄ *s* Lower Upper Minimum Maximum

*H. sapiens* 37 4.2893 0.8765 3.9222 4.6576 2.3576 5.9770

*P. troglodytes* 44 2.6174 0.9918 2.2255 3.0146 1.1186 4.6070

*G. gorilla* 39 3.3482 1.0983 2.9004 3.7812 1.6100 6.1224

**Supplemental Table 3. One-Way Analysis of Variance: Index of Relative AIF Diameter in Hominoids: (AIF Diameter/Geometric Mean)*100***.*

Source Degrees of Sum of Mean F P
 Freedom Squares Square

Among groups 2 72.6781 36.3390 40.97 < .001

Within groups 60 53.2127 .8869

Total 62 125.8907

99% CI for Mean

Taxa n x̄ *s* Lower Upper Minimum Maximum

*H. sapiens* 25 4.6979 1.0691 4.1400 5.3049 2.9070 6.7907

*P. troglodytes* 19 2.1328 0.9345 1.6257 2.7077 0.9999 3.8029

*G. gorilla* 19 3.2384 0.7477 2.7858 3.6626 1.7098 4.8932

**SUPPLEMENT 6: AIF IN SPECIMEN SK-3155**


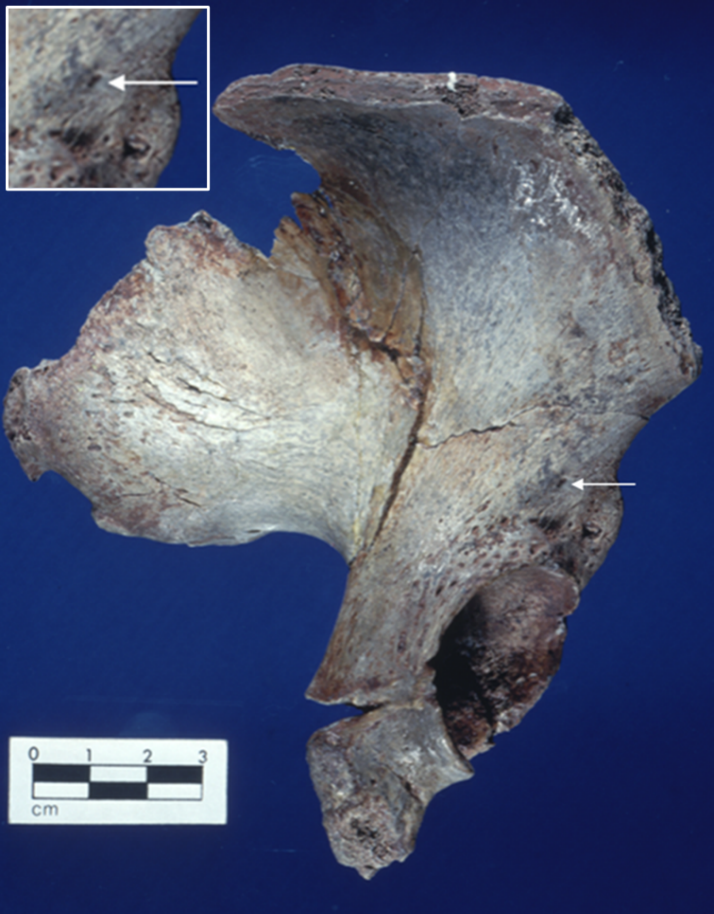


**Supplemental Fig. 5**. SK 3155 displaying a large AIF (arrow). Enlarged image of the AIF (inset). Photo of original specimen courtesy of T.D. White.

**SUPPLEMENT 7. Summary Data**

| **Taxa** | **ID** | **AIF**  **Diam.**  **(mm)** | **NF**  **Diam.**  **(mm)** | **Femur**  **Head**  **Diam.**  **(mm)** | **Humerus**  **Head**  **Diam.**  **(mm)** | **Proximal**  **Tibia**  **Breadth**  **(mm)** | **Distal**  **Humerus**  **Breadth**  **(mm)** | **Acetab**  **Height**  **(mm)** | **Geomet.**  **Mean** |
| --- | --- | --- | --- | --- | --- | --- | --- | --- | --- |
| ***Homo*** |  |  |  |  |  |  |  |  |  |
| *H. sapiens* | 02036 | 2.4 | 1.3 | 45.43 | 45.02 | 72.14 | 56.76 | 51.41 | 0.04461 |
| *H. sapiens* | 00254 | 1.4 | 0.6 | 40.35 | 38.33 | 65.07 | 53.45 | NA | 0.02907 |
| *H. sapiens* | 03236 | 2.8 | 1.2 | 40.48 | 35.32 | 61.83 | 44.60 | NA | 0.06284 |
| *H. sapiens* | 03204 | 3 | 2.5 | 42.06 | 35.86 | 54.60 | 49.98 | NA | 0.06660 |
| *H. sapiens* | 03212 | 2.6 | 0.5 | 44.73 | 43.57 | 70.64 | 56.71 | NA | 0.04919 |
| *H. sapiens* | 03215 | 2.5 | 1.2 | 43.14 | 41.91 | 67.59 | 58.57 | NA | 0.04833 |
| *H. sapiens* | 04005 | 3 | 2.2 | 37.15 | 35.63 | 59.74 | 48.17 | NA | 0.06791 |
| *H. sapiens* | 03302 | 2.5 | 1.2 | 45.70 | 46.26 | 71.41 | 65.49 | NA | 0.04458 |
| *H. sapiens* | 00258 | 1.9 | 1.7 | 38.86 | 37.68 | 66.71 | 53.06 | NA | 0.03982 |
| *H. sapiens* | 04026 | 3 | 2.3 | 42.23 | 39.03 | 69.22 | 54.36 | NA | 0.06012 |
| *H. sapiens* | 01261 | 1.7 | 0.5 | 38.35 | 37.95 | 68.05 | 46.73 | NA | 0.03665 |
| *H. sapiens* | 01004 | 2.6 | 2.3 | 44.20 | 41.31 | 72.50 | 58.19 | NA | 0.04935 |
| *H. sapiens* | 02033 | 1.5 | 1.4 | 40.39 | 37.33 | 67.19 | 49.94 | NA | 0.03163 |
| *H. sapiens* | 04041 | 3 | 3 | 48.50 | 45.26 | 79.09 | 62.21 | NA | 0.05233 |
| *H. sapiens* | 03237 | 1.6 | 1.1 | 44.69 | 43.67 | 75.25 | 59.42 | 52.20 | 0.02944 |
| *H. sapiens* | 04035 | 2.2 | 2.8 | 40.38 | 36.16 | 65.24 | 55.00 | 47.02 | 0.04598 |
| *H. sapiens* | 01272 | 1.7 | 1.7 | 40.37 | 37.83 | 65.62 | 53.13 | 46.59 | 0.03539 |
| *H. sapiens* | 03001 | 2.6 | 1.9 | 43.40 | 40.85 | 73.74 | 56.26 | NA | 0.04993 |
| *H. sapiens* | 02253 | 3 | 1.6 | 48.88 | 45.82 | 73.99 | 66.43 | NA | 0.05208 |
| *H. sapiens* | 02217 | 2.2 | 2 | 48.77 | 46.30 | 75.75 | 59.29 | 54.28 | 0.03899 |
| *H. sapiens* | 02244 | 2.6 | 2.6 | 42.11 | 39.32 | 69.46 | 53.87 | NA | 0.05211 |
| *H. sapiens* | 02609 | 2.7 | 3 | 49.32 | 45.32 | 78.73 | 63.17 | NA | 0.04676 |
| *H. sapiens* | 02250 | 2.2 | 0.5 | 43.04 | 41.95 | 67.68 | 57.32 | NA | 0.04276 |
| *H. sapiens* | 03225 | 2.7 | 1.2 | 40.58 | 34.72 | 62.21 | 56.51 | NA | 0.05723 |
| *H. sapiens* | 03223 | 1.9 | 2 | 39.66 | 38.55 | 60.86 | 50.63 | 43.13 | 0.04078 |
| *H. sapiens* | 00208 | 2.8 | 2.2 | NA | NA | NA | NA | NA | NA |
| *H. sapiens* | 00213L | 1.4 | 1.8 | NA | NA | NA | NA | NA | NA |
| *H. sapiens* | 04044 | 2.8 | 0.8 | NA | NA | NA | NA | 54.19 | NA |
| *H. sapiens* | 00255 | 2 | 0.9 | NA | NA | NA | NA | NA | NA |
| *H. sapiens* | 02025 | 2.2 | 1.9 | NA | NA | NA | NA | NA | NA |
| *H. sapiens* | 02043 | 1.5 | 1.7 | NA | NA | NA | NA | 49.32 | NA |
| *H. sapiens* | 04095 | 2.5 | 2.6 | NA | NA | NA | NA | NA | NA |
| *H. sapiens* | 12215 | 2 | 3 | NA | NA | NA | NA | NA | NA |
| *H. sapiens* | 01010 | 1.7 | 0.5 | NA | NA | NA | NA | 46.11 | NA |
| *H. sapiens* | 06072 | 2.2 | 0.5 | NA | NA | NA | NA | 49.62 | NA |
| *H. sapiens* | 00739 | 3 | 1.2 | NA | NA | NA | NA | 61.89 | NA |
| *H. sapiens* | 31200 | 2.8 | 1.4 | NA | NA | NA | NA | NA | NA |
| *H. sapiens* | 05071 | 1.4 | 0.2 | NA | NA | NA | NA | 48.38 | NA |
| *H. sapiens* | 05025 | 2.1 | 0.6 | NA | NA | NA | NA | NA | NA |
| *H. sapiens* | 01213 | 1.4 | 0.6 | NA | NA | NA | NA | 44.41 | NA |
| *H. sapiens* | 02247 | 2.2 | 1.5 | NA | NA | NA | NA | 51.27 | NA |
| *H. sapiens* | 02006 | 2.5 | 1.7 | NA | NA | NA | NA | 55.83 | NA |
| *H. sapiens* | 01225 | 1.5 | 1.3 | NA | NA | NA | NA | 48.58 | NA |
| *H. sapiens* | 02206 | 2.6 | 1.6 | NA | NA | NA | NA | 43.51 | NA |
| *H. sapiens* | 02055 | 2.1 | 0.8 | NA | NA | NA | NA | 57.23 | NA |
| *H. sapiens* | 01230 | 2.2 | 0.9 | NA | NA | NA | NA | 51.00 | NA |
| *H. sapiens* | 01242 | 3 | 1.9 | NA | NA | NA | NA | 54.37 | NA |
| *H. sapiens* | 00235 | 1.2 | 1.9 | NA | NA | NA | NA | 50.88 | NA |
| *H. sapiens* | 09017 | 2.5 | 1.2 | NA | NA | NA | NA | 49.94 | NA |
| *H. sapiens* | 06006 | 1.9 | 2.3 | NA | NA | NA | NA | 46.36 | NA |
| *H. sapiens* | 05030 | 2.5 | 0.6 | NA | NA | NA | NA | 48.84 | NA |
| *H. sapiens* | 05011 | 2.7 | 1.3 | NA | NA | NA | NA | 52.54 | NA |
| *H. sapiens* | 04907 | 2.2 | 1.9 | NA | NA | NA | NA | 56.46 | NA |
| *H. sapiens* | 05068 | 2.5 | 0.5 | NA | NA | NA | NA | 52.68 | NA |
| *H. sapiens* | 04023 | 2.5 | 1.6 | NA | NA | NA | NA | 50.82 | NA |
| *H. sapiens* | 04029 | 2.8 | 0.5 | NA | NA | NA | NA | 51.91 | NA |
| *H. sapiens* | 04096 | 2.3 | 2.2 | NA | NA | NA | NA | 50.29 | NA |
| *H. sapiens* | 04007 | 1.6 | 1.4 | NA | NA | NA | NA | 51.90 | NA |
| *H. sapiens* | 04016 | 2.8 | 0.5 | NA | NA | NA | NA | 55.10 | NA |
| *H. sapiens* | 04088 | 2.5 | 1.8 | NA | NA | NA | NA | 53.18 | NA |
| *H. sapiens* | 03269 | 1.7 | 0.5 | NA | NA | NA | NA | 53.22 | NA |
| *H. sapiens* | 03241 | 1.9 | 0.5 | NA | NA | NA | NA | 48.23 | NA |
| *H. sapiens* | 03216 | 2.8 | 0.5 | NA | NA | NA | NA | 54.46 | NA |
| *H. sapiens* | 03221 | 2.6 | 2.9 | NA | NA | NA | NA | 49.80 | NA |
| *H. sapiens* | 03276 | 2.5 | 0.7 | NA | NA | NA | NA | NA | NA |
| ***Pan*** |  |  |  |  |  |  |  |  |  |
| *P. troglodytes* | 2747 | 1.7 | 1.4 | 32.90 | 37.96 | 54.13 | 59.07 | 38.60 | 0.03803 |
| *P. troglodytes* | 1882 | 0.9 | 0.5 | 32.71 | 40.73 | 55.31 | 60.07 | 38.40 | 0.01962 |
| *P. troglodytes* | 1880 | 1.5 | 1 | 35.84 | 43.97 | 61.23 | 67.97 | 42.01 | 0.02964 |
| *P. troglodytes* | 1843 | 0.5 | 0.5 | 33.58 | 34.93 | 58.04 | 62.63 | 37.59 | 0.01100 |
| *P. troglodytes* | 3551 | 0.9 | 0.5 | 28.75 | 32.20 | 49.66 | 54.39 | 33.11 | 0.02263 |
| *P. troglodytes* | 2746 | 0.5 | 0.5 | 32.95 | 36.80 | 54.72 | 57.65 | 38.40 | 0.01131 |
| *P. troglodytes* | 1758 | 0.7 | 0.8 | 35.95 | 39.96 | 60.28 | 64.23 | 42.49 | 0.01441 |
| *P. troglodytes* | 3539 | 1.5 | 0.5 | 32.10 | 36.16 | 53.64 | 60.40 | NA | 0.03406 |
| *P. troglodytes* | 2072 | 1.8 | 1.3 | 35.44 | 39.07 | 58.23 | 64.04 | 42.48 | 0.03776 |
| *P. troglodytes* | 1718 | 0.6 | 1 | 32.27 | 38.85 | 57.06 | 61.70 | 36.02 | 0.01309 |
| *P. troglodytes* | 2771 | 0.5 | 1.8 | 29.22 | 30.92 | 54.12 | 56.24 | 34.40 | 0.01228 |
| *P. troglodytes* | 2748 | 1.5 | 1.1 | 36.83 | 38.55 | 62.80 | 68.12 | 42.41 | 0.03022 |
| *P. troglodytes* | 3538 | 0.9 | 0.6 | 33.29 | 36.31 | 59.92 | 56.04 | NA | 0.02005 |
| *P. troglodytes* | 1726 | 1.1 | 2 | 36.83 | 41.94 | 62.22 | 68.92 | 42.19 | 0.02168 |
| *P. troglodytes* | 1724 | 1.2 | 0.5 | 32.81 | 38.19 | 64.72 | 58.06 | 35.50 | 0.02576 |
| *P. troglodytes* | 1056 | 1.3 | 0.5 | 35.44 | 40.72 | 63.44 | 74.47 | 41.02 | 0.02544 |
| *P. troglodytes* | 1713 | 0.8 | 0.8 | 31.25 | 35.73 | 56.77 | 59.25 | 36.50 | 0.01817 |
| *P. troglodytes* | 1766 | 0.5 | 1.4 | 35.88 | 37.68 | 62.94 | 71.37 | 43.23 | 0.01007 |
| *P. troglodytes* | 1762 | 0.5 | 0.9 | 35.86 | 38.59 | 64.13 | 70.46 | 44.70 | 0.01000 |
| *P. troglodytes* | 1721 | 1.1 | 0.8 | NA | NA | NA | NA | 38.30 | NA |
| *P. troglodytes* | 1755 | 0.9 | 0.9 | NA | NA | NA | NA | 36.89 | NA |
| *P. troglodytes* | 0629 | 1.5 | 1.2 | NA | NA | NA | NA | 39.10 | NA |
| *P. troglodytes* | 0543 | 1.5 | 1.2 | NA | NA | NA | NA | 42.02 | NA |
| *P. troglodytes* | 0171 | 1.2 | 1 | NA | NA | NA | NA | 38.10 | NA |
| *P. troglodytes* | 1706 | 1 | 0.6 | NA | NA | NA | NA | 39.59 | NA |
| *P. troglodytes* | 1707 | 0.8 | 0.8 | NA | NA | NA | NA | 37.82 | NA |
| *P. troglodytes* | 1708 | 0.6 | 0.8 | NA | NA | NA | NA | 41.50 | NA |
| *P. troglodytes* | 1722 | 0.6 | 1.7 | NA | NA | NA | NA | 44.90 | NA |
| *P. troglodytes* | 1737 | 1.4 | 1.5 | NA | NA | NA | NA | 34.03 | NA |
| *P. troglodytes* | 1738 | 0.8 | 0.5 | NA | NA | NA | NA | 43.50 | NA |
| *P. troglodytes* | 1741 | 0.9 | 1.8 | NA | NA | NA | NA | 36.29 | NA |
| *P. troglodytes* | 1744 | 1 | 0.5 | NA | NA | NA | NA | 36.30 | NA |
| *P. troglodytes* | 1745 | 0.9 | 0.5 | NA | NA | NA | NA | 39.00 | NA |
| *P. troglodytes* | 1748 | 0.5 | 0.5 | NA | NA | NA | NA | 37.50 | NA |
| *P. troglodytes* | 1749 | 1.2 | 0.8 | NA | NA | NA | NA | 41.63 | NA |
| *P. troglodytes* | 1759 | 1.7 | 0.5 | NA | NA | NA | NA | 36.90 | NA |
| *P. troglodytes* | 1763 | 0.9 | 0.5 | NA | NA | NA | NA | 36.22 | NA |
| *P. troglodytes* | 1769 | 1 | 1.7 | NA | NA | NA | NA | 37.70 | NA |
| *P. troglodytes* | 1770 | 0.8 | 1.7 | NA | NA | NA | NA | 35.04 | NA |
| *P. troglodytes* | 1775 | 0.7 | 1.8 | NA | NA | NA | NA | 39.70 | NA |
| *P. troglodytes* | 2026 | 0.7 | 1.8 | NA | NA | NA | NA | 41.81 | NA |
| *P. troglodytes* | 2027 | 1.4 | 1.1 | NA | NA | NA | NA | 39.92 | NA |
| *P. troglodytes* | 2071 | 1 | 1.6 | NA | NA | NA | NA | 43.40 | NA |
| *P. troglodytes* | 2823 | 1.5 | 0.9 | NA | NA | NA | NA | 39.12 | NA |
| *P. troglodytes* | 1723 | 1.1 | 2 | NA | NA | NA | NA | 41.58 | NA |
| *P. troglodytes* | 1720 | 1.6 | 0.5 | NA | NA | NA | NA | 35.12 | NA |
| ***Gorilla*** |  |  |  |  |  |  |  |  |  |
| *G. gorilla* | 3556 | 2.5 | 1.3 | NA | NA | NA | NA | NA | NA |
| *G. gorilla* | 1730 | 2 | 2 | 51.47 | 62.81 | 88.77 | 94.96 | 56.40 | 0.02768 |
| *G. gorilla* | 1733 | 2.7 | 2.3 | 51.80 | 61.77 | 91.31 | 94.96 | 59.50 | 0.03720 |
| *G. gorilla* | 1731 | 2 | 2.9 | 46.40 | 53.73 | 81.06 | 95.84 | 52.58 | 0.03015 |
| *G. gorilla* | 1729 | 1.2 | 1.8 | 47.54 | 55.57 | 87.86 | 104.54 | 54.42 | 0.01710 |
| *G. gorilla* | 1764 | 1.2 | 0.7 | 37.22 | 46.74 | 65.13 | 73.00 | 44.30 | 0.02238 |
| *G. gorilla* | 1765 | 1.9 | 1.9 | 38.39 | 43.03 | 66.33 | 72.17 | 46.52 | 0.03583 |
| *G. gorilla* | 1859 | 2.9 | 2 | 50.51 | 66.10 | 86.81 | 102.35 | 54.60 | 0.03930 |
| *G. gorilla* | 1408 | 1.9 | 1.1 | 50.97 | 56.69 | 94.75 | 98.56 | 57.59 | 0.02636 |
| *G. gorilla* | 3400 | 3 | 3 | 52.82 | 58.65 | 289 | 91.69 | NA | 0.03152 |
| *G. gorilla* | 3397 | 1.8 | 2.2 | 51.77 | 58.29 | 78.9 | 92.36 | NA | 0.02629 |
| *G. gorilla* | 3393 | 2.6 | 1.8 | 38.82 | 45.14 | 62.58 | 72.69 | NA | 0.04893 |
| *G. gorilla* | 1020 | 2.5 | 2 | 50.67 | 57.48 | 86.09 | 100.55 | 56.22 | 0.03528 |
| *G. gorilla* | 1407 | 2.7 | 2 | 51.71 | 57.90 | 87.77 | 110.63 | 64.50 | 0.03677 |
| *G. gorilla* | 1416 | 1.8 | 1.3 | 49.14 | 56.52 | 89.46 | 104.68 | 56.60 | 0.02521 |
| *G. gorilla* | 2739 | 3 | 1.3 | 50.98 | 56.83 | 85.15 | 101.94 | NA | 0.04236 |
| *G. gorilla* | 2741 | 2.6 | 1.2 | 54.77 | 61.04 | 90.36 | 97.53 | NA | 0.03529 |
| *G. gorilla* | 2767 | 2.2 | 2.8 | 49.94 | 55.70 | 80.29 | 97.11 | NA | 0.03224 |
| *G. gorilla* | 2742 | 2 | 1.4 | 38.59 | 48.22 | 63.46 | 77.18 | NA | 0.03640 |
| *G. gorilla* | 1953 | 1.6 | 1.9 | 41.32 | 43.45 | 67.95 | 75.58 | NA | 0.02904 |
| *G. gorilla* | 1425 | 1.1 | 0.5 | NA | NA | NA | NA | 58.39 | NA |
| *G. gorilla* | 1423 | 1.8 | 1.1 | NA | NA | NA | NA | 49.60 | NA |
| *G. gorilla* | 1736 | 1.7 | 1.7 | NA | NA | NA | NA | 61.53 | NA |
| *G. gorilla* | 1431 | 1.6 | 1.9 | NA | NA | NA | NA | 58.30 | NA |
| *G. gorilla* | 1728 | 1.1 | 1.2 | NA | NA | NA | NA | 61.01 | NA |
| *G. gorilla* | 1756 | 1 | 2.4 | NA | NA | NA | NA | 47.57 | NA |
| *G. gorilla* | F627 | 1 | 2.2 | NA | NA | NA | NA | 47.02 | NA |
| *G. gorilla* | F626 | 2 | 1.8 | NA | NA | NA | NA | 45.90 | NA |
| *G. gorilla* | 1704 | 1.1 | 1.8 | NA | NA | NA | NA | 46.46 | NA |
| *G. gorilla* | 1709 | 1.8 | 1.3 | NA | NA | NA | NA | 59.00 | NA |
| *G. gorilla* | 1430 | 1.3 | 1.4 | NA | NA | NA | NA | 58.77 | NA |
| *G. gorilla* | 1419 | 2.7 | 1.1 | NA | NA | NA | NA | 44.11 | NA |
| *G. gorilla* | 1417 | 1.7 | 2.3 | NA | NA | NA | NA | 51.80 | NA |
| *G. gorilla* | 1732 | 0.9 | 1.5 | NA | NA | NA | NA | 55.87 | NA |
| *G. gorilla* | 1717 | 2 | 1.9 | NA | NA | NA | NA | 56.23 | NA |
| *G. gorilla* | 1712 | 2.4 | 2.4 | NA | NA | NA | NA | 57.38 | NA |
| *G. gorilla* | 1780 | 1.7 | 1.9 | NA | NA | NA | NA | 58.30 | NA |
| *G. gorilla* | 1754 | 2.2 | 1.2 | NA | NA | NA | NA | 57.04 | NA |
| *G. gorilla* | 1752 | 1.2 | 1.8 | NA | NA | NA | NA | 51.28 | NA |
| *G. gorilla* | 1751 | 2.1 | 1 | NA | NA | NA | NA | 47.30 | NA |
| *G. gorilla* | 1743 | 1.3 | 1.1 | NA | NA | NA | NA | 46.33 | NA |
| *G. gorilla* | 1797 | 1.2 | 2 | NA | NA | NA | NA | 56.87 | NA |
| *G. gorilla* | 1798 | 2.7 | 1.8 | NA | NA | NA | NA | 48.52 | NA |
| *G. gorilla* | 1796 | 2.2 | 1.2 | NA | NA | NA | NA | 50.40 | NA |
| *G. gorilla* | 1795 | 1.3 | 0.5 | NA | NA | NA | NA | 56.89 | NA |
| *G. gorilla* | 1794 | 1.6 | 1.9 | NA | NA | NA | NA | 45.03 | NA |
| *G. gorilla* | 1787 | 1.5 | 1.6 | NA | NA | NA | NA | 54.30 | NA |
| *G. gorilla* | 1801 | 2 | 1.8 | NA | NA | NA | NA | 46.07 | NA |
| ***Pongo*** |  |  |  |  |  |  |  |  |  |
| *P. pygmaeus* | 1055 | 0.6 | 0.6 | 32.63 | 37.83 | 52.87 | 58.7 | 37.52 | 0.01356 |
| *P. pygmaeus* | 623 | 0.8 | 1.1 | 36.84 | 44.08 | 61.19 | 71.01 | 44.00 | 0.01552 |
| *P. pygmaeus* | 1030 | 0.5 | 0.9 | NA | NA | NA | NA | 33.60 | NA |
| *P. pygmaeus* | 1167 | 1 | 0.9 | NA | NA | NA | NA | 38.42 | NA |
| *P. pygmaeus* | 15/8 T | 1 | 0.9 | NA | NA | NA | NA | NA | NA |
| *P. pygmaeus* | 1168 | 0.5 | 1.9 | NA | NA | NA | NA | NA | NA |
| *P. pygmaeus* | 172 | NA | NA | 40.28 | 48.32 | 63.52 | 70.77 | 45.40 | NA |
| ***Hylobates*** |  |  |  |  |  |  |  |  |  |
| *H. lar* | 3903 | Absent | NA | NA | NA | NA | NA | 18.70 | NA |
| *H. lar* | 3905 | Absent | NA | NA | NA | NA | NA | 20.41 | NA |
| *H. lar* | 3902 | Absent | NA | NA | NA | NA | NA | 19.32 | NA |
| *H. lar* | 3898 | Absent | NA | NA | NA | NA | NA | 20.58 | NA |
| *H. lar* | 3896 | Absent | NA | NA | NA | NA | NA | 19.00 | NA |
| *H. lar* | 3893 | Absent | NA | NA | NA | NA | NA | 20.50 | NA |
| *H. lar* | 3890 | Absent | NA | NA | NA | NA | NA | 17.81 | NA |
| *H. lar* | 3888 | Absent | NA | NA | NA | NA | NA | 20.00 | NA |
| *H. lar* | 3887 | Absent | NA | NA | NA | NA | NA | 20.90 | NA |
| *H. lar* | 3886 | Absent | NA | NA | NA | NA | NA | 19.52 | NA |
| *H. lar* | 3885 | Absent | NA | NA | NA | NA | NA | 21.03 | NA |
| *H. lar* | 3884 | Present | NA | NA | NA | NA | NA | 20.90 | NA |
| *H. lar* | 3883 | Absent | NA | NA | NA | NA | NA | 20.29 | NA |
| *H. agilis* | 1048 | Present | NA | NA | NA | NA | NA | 17.90 | NA |
| *H. lar* | 3904 | Absent | NA | NA | NA | NA | NA | 18.48 | NA |
| *H. lar* | 3881 | Absent | NA | NA | NA | NA | NA | 18.87 | NA |
| *H. lar* | 3900 | Absent | NA | NA | NA | NA | NA | 17.90 | NA |
| *H. concolor* | 0161 | Absent | NA | NA | NA | NA | NA | NA | NA |
| *H. lar* | 3882* | Absent | NA | NA | NA | NA | NA | NA | NA |
| *H. lar* | 3880 | Absent | NA | NA | NA | NA | NA | NA | NA |
| *H. lar* | 3912 | Absent | NA | NA | NA | NA | NA | NA | NA |
| *H. lar* | 3911 | Absent | NA | NA | NA | NA | NA | NA | NA |
| *H. lar* | 3910 | Present | NA | NA | NA | NA | NA | NA | NA |
| *H. lar* | 3909 | Absent | NA | NA | NA | NA | NA | NA | NA |
| *H. lar* | 3908 | Absent | NA | NA | NA | NA | NA | NA | NA |
| *H. lar* | 3907 | Absent | NA | NA | NA | NA | NA | NA | NA |
| *H. lar* | 3879 | Absent | NA | NA | NA | NA | NA | NA | NA |
| *H. lar* | 3878 | Present | NA | NA | NA | NA | NA | NA | NA |
| *H. lar* | 3895 | Absent | NA | NA | NA | NA | NA | NA | NA |
| *H. lar* | 3894 | Absent | NA | NA | NA | NA | NA | NA | NA |
| *H. lar* | 3892 | Absent | NA | NA | NA | NA | NA | NA | NA |
| *H. lar* | 3891* | Absent | NA | NA | NA | NA | NA | NA | NA |
| *H. lar* | 3890 | Absent | NA | NA | NA | NA | NA | NA | NA |
| *H. lar* | 3889 | Absent | NA | NA | NA | NA | NA | NA | NA |
| *H. lar* | 3897 | Absent | NA | NA | NA | NA | NA | NA | NA |
| *H. lar* | 3899 | Present | NA | NA | NA | NA | NA | NA | NA |
| *H. lar* | 3900 | Absent | NA | NA | NA | NA | NA | NA | NA |
| *H. lar* | 3901 | Absent | NA | NA | NA | NA | NA | NA | NA |
| *H. lar* | 3906 | Absent | NA | NA | NA | NA | NA | NA | NA |
| *H. concolor* | 16528 | Absent | NA | NA | NA | NA | NA | NA | NA |

*Note: Specimens that are late subadult but have attained full adult size and fusion.
